# Anti-Hyperglycaemic, Hepatoprotective, and Nephroprotective Effects of *Wrightia tinctoria* Seeds and *Enterococcus ratti* in Alloxan-Induced Diabetic Mice

**DOI:** 10.3390/ijms27198792

**Published:** 2026-09-30

**Authors:** Mohsin Munawar, Syed Turab Raza

**Affiliations:** 1 State Key Laboratory of Vegetation Structure, Functions and Construction, and Yunnan Key Laboratory of Soil Erosion Prevention and Green Development, Yunnan University, Kunming 650500, China; f2202500016@stu.ynu.edu.cn; 2 Yunnan Key Laboratory of Meteorological Disasters and Climate Resources in the Greater Mekong Sub Region, School of Earth Sciences, Yunnan University, Kunming 650500, China

**Keywords:** *Wrightia tinctoria*, diabetes mellitus, nephroprotective effects, medicinal plants, probiotics

## Abstract

Diabetes mellitus (DM), a chronic metabolic disease that leads to damage to multiple organ systems, needs to be investigated in natural ways. The present experiment was performed to determine the anti-hyperglycaemic, nephroprotective and hepatoprotective potential of the methanolic extract of *Wrightia tinctoria* (*W. tinctoria)* seeds, and *Enterococcus ratti* (*E. ratti*), in alloxan-induced hyperglycaemic mice (150 mg/kg body weight). *W. tinctoria* and *E. ratti* were screened out from different locally medicinally important plants and bacterial strains by different in vitro tests. *W. tinctoria* seeds showed α-amylase inhibition through the starch–iodine test (88.64% at 100 mg/mL), inhibition of glycosylation of haemoglobin (88.34% at 3.56 mg/mL), and an inhibitory effect on glucose uptake by yeast cells (91.62% at 500 µg/mL). Among various bacterial strains used for in vitro screening, *E. ratti* exhibited the most effective glucose adsorption capacity (26.2% at colony-forming unit (CFU) 10^11^). High-performance liquid chromatography (HPLC) and Fourier-transformed infrared (FT-IR) spectroscopy analyses provided preliminary information on the phytochemical composition of the extract. In vivo administration of *W. tinctoria* (200, 400 mg/kg body weight), *E. ratti* (60 µL at a CFU 10^11^) alone and their combination produced significant dose-dependent improvements in treatment groups, with the combination demonstrating enhanced efficacy compared to individual treatments. Future research should focus on detailed mechanisms underlying these protective effects using transcriptomic and proteomic approaches and developing standardized formulation strategies for translation into clinical applications.

## 1. Introduction

Diabetes mellitus (DM), a metabolic disorder, is a major health issue globally [1,2,3,4]. It is characterized by excessive blood sugar levels, and a growing epidemic worldwide, posing significant financial and healthcare policy challenges [5,6]. It severely impacts individuals, families, and society worldwide [7]. According to International Diabetes Federation (IDF) reports, 537 million people had diabetes in 2021, and this number may increase to 643 and 783 million by 2030 and 2045, respectively [8,9]. In 2015, the disease caused 1.6 million deaths and remains particularly prevalent in underdeveloped and developing countries [10]. Pakistan has been ranked as the third-largest population of people with diabetes, only after China and India [11]. High levels of sugar in the blood can cause damage to the kidneys, heart attacks, strokes, lower limb amputation and diabetic foot ulcers [12,13]. An imbalance between reactive oxygen species (ROS) formation and antioxidant defence systems contributes to the pathogenesis of diabetes mellitus [14,15].

The Food and Drug Administration (FDA) has approved synthetic medications such as metformin, pycnogenol, acarbose, miglitol, voglibose and glipizide, which are used to lower blood glucose levels, but they have numerous side effects such as nausea, bloating, diarrhoea, B12 deficiency, muscle pain and upset stomach [16,17,18]. Drug resistance may also be developed due to their constant and irregular use [19]. Due to certain demerits of conventional medications, the global scientific community is shifting its research from synthetic drugs to natural-ingredient-based remedies. In this regard, various natural herbs, probiotics and medicinal plants have been used to treat diabetes, which subsequently improves blood insulin levels. For health care, almost 80% of the world’s population depends upon herbal drugs [20]. Researchers have evaluated the potential of these herbs, probiotics, and plants experimentally, and clinical trials have also shown their efficacy [21]. Probiotics exhibit positive effects on lowering glucose levels [22,23]. Numerous positive effects of probiotics on human health have been observed, including immunomodulation and reduction in serum cholesterol and triglycerides. A change in the gut microbiota could improve metabolic control in individuals with type 2 diabetes (T2DM) [24] and enhance gut-barrier function, as observed in genetically obese mice. Thus, these bioactive compounds may potentially prevent and treat diabetes [25].

Various plants have shown antidiabetic activity. *Adhatoda vesica*, locally known as Barg e Bansa, is a medicinal plant with various health benefits. Its leaf extract contains alkaloids, phenols and flavonoids, which contribute to treating various biological disorders, including antidiabetic properties [26]. *Calendula officinalis* (commonly known as pot marigold) exhibits many pharmacological and therapeutic activities because of these active ingredients: sterols, saponins, flavonoids, amino acids, aromatics, carbohydrates, phenolic acids, carotenoids, and minerals [27,28]. These ingredients possess anti-HIV, antispasmolytic, analgesic, hepatoprotective, uterotonic, antipyretic, diuretic, and antidiabetic properties [29]. *Saraca indica*, (commonly known as ashoka or asoka) [30] is valued for its therapeutic potential because of the phenolic, tannins, flavonoids, leucocyanidin, procyanidin p2, and catechins content in its bark and leaves [31]. Its bioactive compounds offer antibacterial, antioxidant, anticancer, antidepressant, analgesic, diuretic, antipyretic, and antidiabetic potential [32]. Similarly, *Wrightia tinctoria* (commonly known as sweet indrajao) contains flavonoids, phenols, saponins, and various other chemical components in its different parts. The plant has therapeutic and pharmaceutical properties and is commonly used to treat skin diseases, tooth decay, wound injury, inflammation, diabetes, viral and fungal diseases, hypoglycaemia, oxidative stress and diarrhoea. *Ziziphus jujuba* (commonly known as red date or jujube) [33] contains bioactive components that are highly significant, i.e., flavonoids, phenolic acids, triterpenoids, carotenoids and organic acids [34]. These bioactive ingredients contribute to vast medicinal properties, including anti-inflammatory, antidiabetic, antioxidant, antiapoptotic and anti-hyperlipidaemia [35,36]. Moreover, *Enterococcus ratti* MF183967, a lactic acid bacterium (LAB), contributes positively to food fermentation and characterized for its antimicrobial potential [37,38,39]. Recent evidence has demonstrated that the plant-probiotic combinations may offer superior therapeutic outcomes [40]. Probiotic strains enhance the bioavailability of plant derived polyphenols through enzymatic hydrolysis of glycosides into more absorbable aglycones [41]. Moreover, polyphenols from medicinal plants can selectively promote the growth of beneficial gut microbiota, creating a synergistic relationship.

The plants evaluated in this study were selected based on the documented ethno-medicinal use. However, comparative evaluation of these medicinal plants under identical experimental conditions remains limited. To achieve valuable, cost-effective results, we used various in vitro screening methods to identify the most effective plant extract traditionally used to manage the hyperglycaemic response. Since diabetes is a multifactorial metabolic disorder, no single in vitro assay can predict the antidiabetic efficacy of natural products. Therefore, a series of biochemical assays were used in this study as preliminary screening tools to evaluate different aspects of anti-hyperglycaemic potential, including inhibition of carbohydrate digestion, antiglycation activity and glucose consumption by probiotics. These assays were intended only for the initial screening and for in vivo evaluation of the best-performing plant and LAB strain with probiotic potential. Animal models are essential for preclinical evaluation of potential antidiabetic agents. The alloxan-induced diabetes model is widely used due to simplicity, reproducibility, and low cost. This model is valuable for screening agents with potential β-cell protective properties. It does not replicate the insulin resistance, obesity, or progressive β-cell dysfunction characteristics of T2DM. Therefore, our findings should be interpreted as preliminary evidence of potential antidiabetic activity in an insulin-deficient state, rather than direct evidence of efficacy in T2DM.

The proposed research plan intends to study the influence of herbal extracts of *W. tinctoria*, and *E. ratti* in ameliorating alloxan-induced hyperglycaemia and its associated complications in mice. The products’ methanolic extracts contain antidiabetic compounds such as flavonoids, tannins, phenolics and alkaloids that enhance pancreatic tissue function. The aim of this proposed study is to evaluate and compare the individual and combined role of the selected plant extract and bacteria in artificially induced diabetes in mice. The use of these plants may result in reduced toxicity and side effects, ultimately helping to treat such metabolic diseases in the future.

## 2. Results

### 2.1. In Vitro Screening Tests

**Figure 1:**
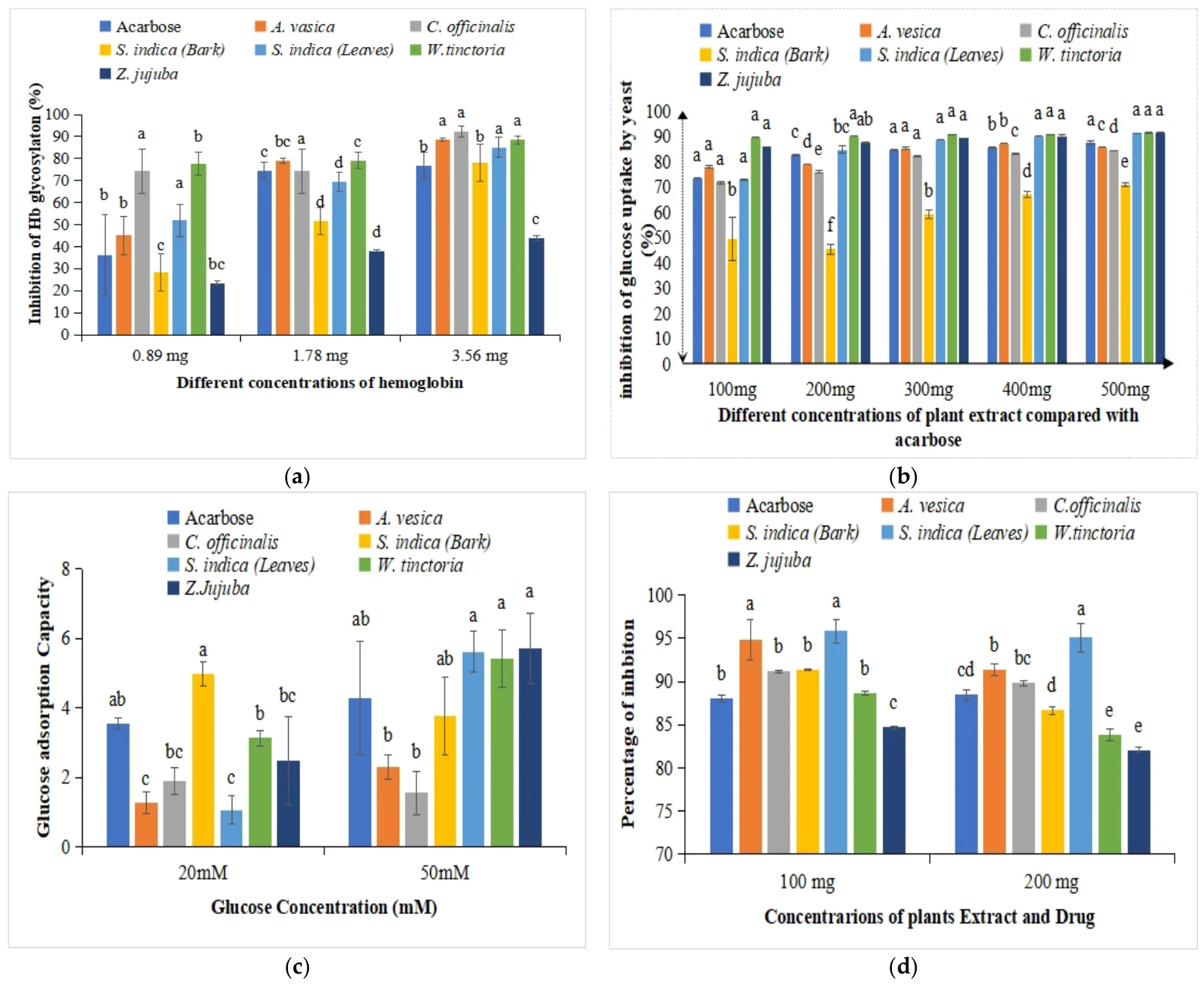
Effects of methanolic extracts of selected plants and acarbose dissolved in dimethyl sulfoxide (DMSO) on (**a**) inhibition of Hb glycosylation, (**b**) glucose uptake by yeast cells, (**c**) glucose adsorption capacity, and (**d**) inhibition of α-amylase. The error bar shows standard deviations (S.D) within the treatments. Different letters above bars indicate statistically significant differences.

A non-enzymatic glycosylated haemoglobin (Hb) assay was used to determine the percentage inhibition of Hb glycosylation. The percentage inhibition in glycosylated Hb was concentration-dependent. Among methanolic extracts, the 3.56 mg/mL concentrations of *A. vesica*, *C. officinalis*, and *W. tinctoria* showed 88.50%, 92.25%, and 88.34% inhibition, respectively (Figure 1a). The concentration of glucose in the solution after a specific incubation period served as an indicator of glucose uptake by yeast cells. Glucose uptake was dependent on the concentration of the plant extract (100–500 µg). Among plant extracts, *S. indica*, *W. tinctoria* and *Z. jujuba* showed percentage inhibitions of glucose by yeast cells of 91.50%, 91.62% and 91.71%, respectively (Figure 1b). In our experiment, *S. indica*, *W. tinctoria* and *Z. jujuba* showed better results (5.61, 5.40 and 5.71 moles, respectively) when compared to the standard drug at 50 mM concentration (Figure 1c). The methanolic extracts of plants have shown the ability to inhibit α amylase as well. Among the plants, *S. indica* and *W. tinctoria* (95.09% and 88.64% respectively) showed significantly higher α-amylase inhibitory activity than the other plants at 100 mg/mL concentration (Figure 1d). Based on consistently better performance across all in vitro assays, *W. tinctoria* was selected for in vivo evaluation. Among all plants, *W. tinctoria* extract met the pre-defined criteria (≥85% result in all assays). The anti-hyperglycaemic potential of selected bacterial strains was determined by in vitro glucose adsorption capacity assay. It was noted that all strains had significant results, but *E. ratti* had the highest glucose adsorption capacity (26.2%).

### 2.2. Hypoglycaemic, Hepatoprotective and Nephroprotective Response of W. tinctoria and E. ratti in Alloxanized Mice

#### 2.2.1. Determination of Fasting Blood Glucose (FBG) Level

**Figure 2:**
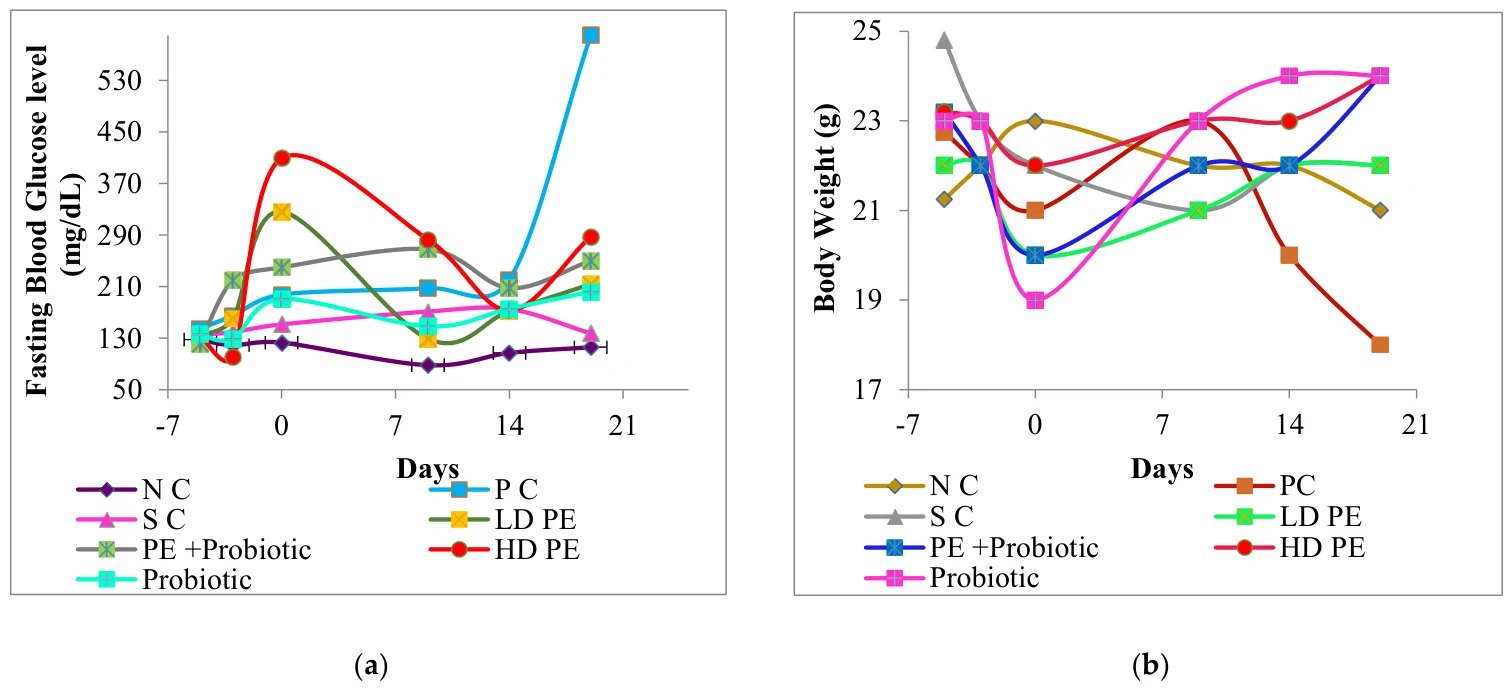
Effects of methanolic extract of *W. tinctoria* (seeds), and *E. ratti* on (**a**) blood glucose level and (**b**) body weight in treated groups, and compared with control groups. Measurements were taken at baseline (day 7), after diabetes induction (day 0) and weekly during treatment (days 7, 14 and 21).

The FBG level was elevated in all groups after diabetes induction (≥250 mg/dL). The FBG level of the PE + Probiotic group treated with a combination of seeds of *W. tinctoria* (200 mg/kg body weight) and *E. ratti* significantly decreased to normal levels by day 21. The other groups treated with plant extract and probiotics showed a progressive reduction in FBG over the 21-day treatment period, although the degree of reduction varied between groups (Figure 2a).

#### 2.2.2. Weight of Mice

The weight of mice was measured before the start of the experiment (baseline), and weekly thereafter. Following diabetes induction, all groups showed a reduction in weight compared to baseline. However, upon initiation of treatment, the body weight of treated groups recovered progressively over the 21-day period, except in the PC group, which continued to lose weight due to diabetes. The best weight recovery was observed in the probiotic group, followed by the HD PE and PE + Probiotic groups. However, there was no significant difference between the weight of the treatment groups’ mice and the SC group (Figure 2b).

#### 2.2.3. Blood Biomarkers

**Figure 3:**
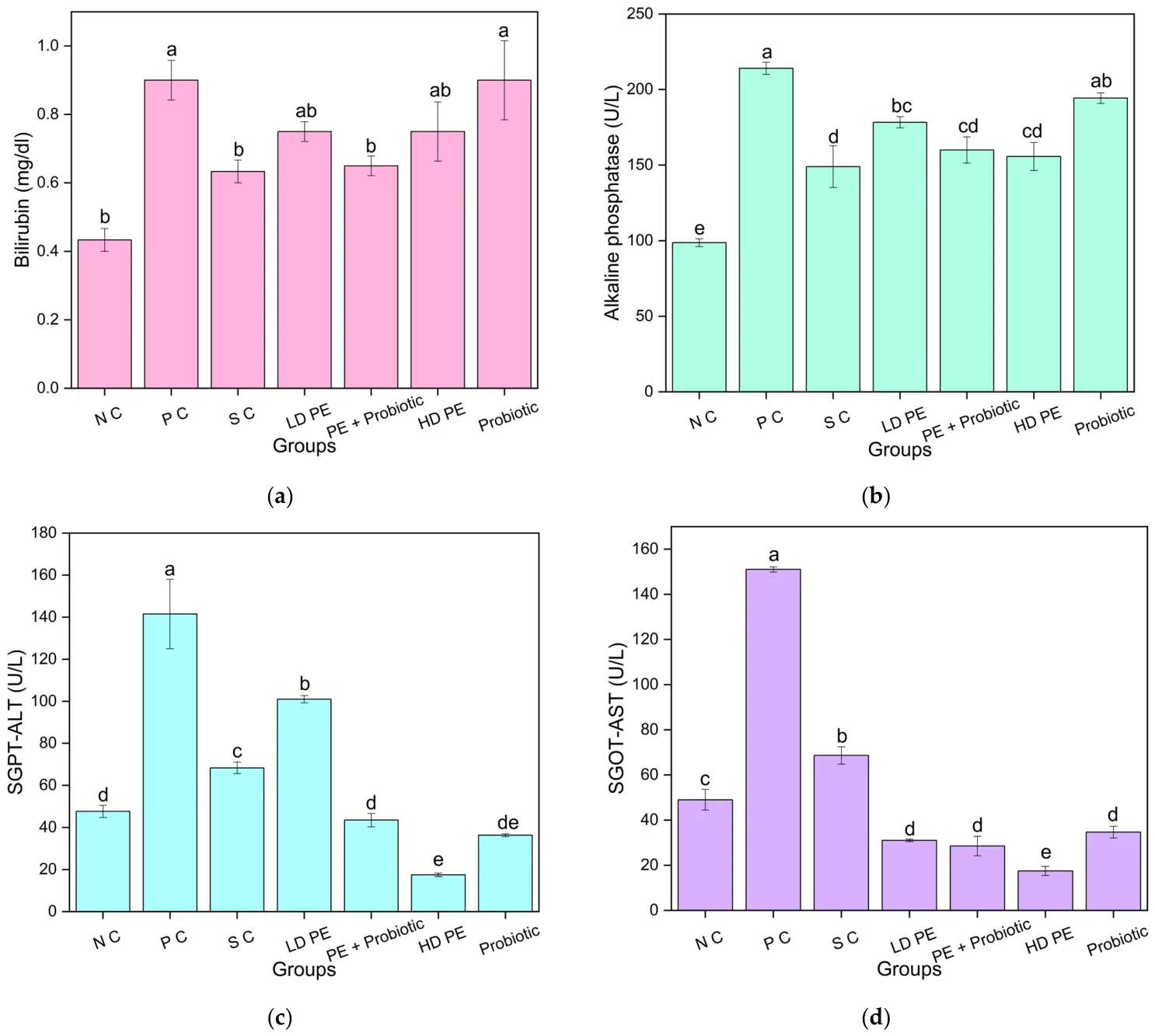
Effects of methanolic extract of *W. tinctoria* (seeds), *E. ratti* and the combination of both on liver function test, including (**a**) bilirubin, (**b**) ALP, (**c**) AST/SGOT and (**d**) ALT/SGPT levels in treated groups and compared with control groups. The error bar shows standard deviations (S.D) within the treatments. Different letters above bars indicate statistically significant differences.

Bilirubin, alkaline phosphatase (ALP), alanine aminotransferase (ALT), and aspartate aminotransferase (AST) levels were high in the diabetic mice. However, they were significantly decreased in the experimental groups and more pronounced in those treated with a high dose of *W. tinctoria* (400 mg/kg body weight) (Figure 3a–d).

**Figure 4:**
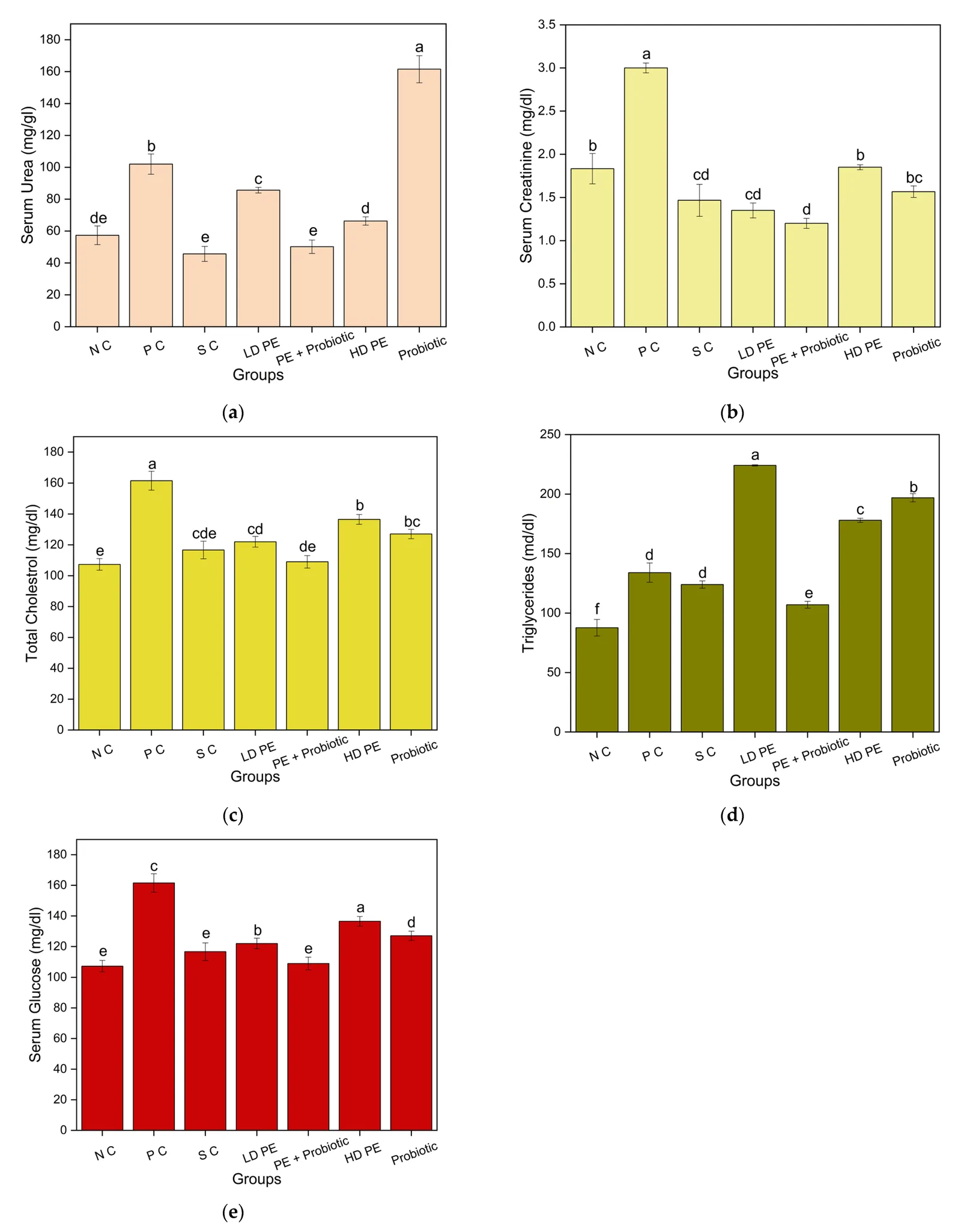
Effects of methanolic extract of *W. tinctoria* (seeds), *E. ratti* and the combination of both on renal function test, including (**a**) urea and (**b**) creatinine, lipid profile, including (**c**) cholesterol, (**d**) triglycerides, and (**e**) serum glucose in treated groups and compared with control groups. The error bar shows standard deviations (S.D) within the treatments. Different letters above bars indicate statistically significant differences.

In the same manner, the group treated with the methanolic extract of the plants and LAB showed a decreasing trend of urea, creatinine levels, and lipid profile (total cholesterol and triglyceride levels) as compared to the PC group (Figure 4a,b).

Serum glucose level was increased in the untreated alloxanized group but reversed to normal in the treated groups (Figure 4). One-way analysis of variance (ANOVA) demonstrated the significant difference among the experimental groups as bilirubin (*F*_6,28_ = 6.70; *p* = 0.0017), ALP (*F*_6,28_ = 23.90; *p* = 0.0001), ALT (*F*_6,28_ = 42.65; *p* = 0.0001), AST (*F*_6,28_ = 218.07; *p* = 0.0001), urea (*F*_6,28_ = 55.9; *p* = 0.0001), creatinine (*F*_6,28_ = 29.58; *p* = 0.0001), serum glucose (*F*_6,28_ = 99.05; *p* = 0.0001), total cholesterol (*F*_6,28_ = 22.77; *p* = 0.0001) and triglycerides (*F*_6,28_ = 5.74; *p* = 0.0037).

The further analysis of quantification of *W. tinctoria* seeds by high-performance liquid chromatography (HPLC) and Fourier-transform infrared (FT-IR) spectroscopy are presented in Supplementary Materials (Figures S1 and S2 and Table S1). The HPLC chromatogram of the methanolic extract showed the presence of highly polar and hydrophilic compounds. FT-IR spectroscopy confirmed the presence of functional groups characteristic of polyphenols (O-H stretching, ~3390 cm^−1^) and aliphatic compounds (C-H stretching, ~2292 cm^−1^). These results provide supporting evidence for the presence of various functional groups but do not constitute definitive compound identification.

### 2.3. Histological Analysis of Pancreas, Liver and Kidney

**Figure 5:**
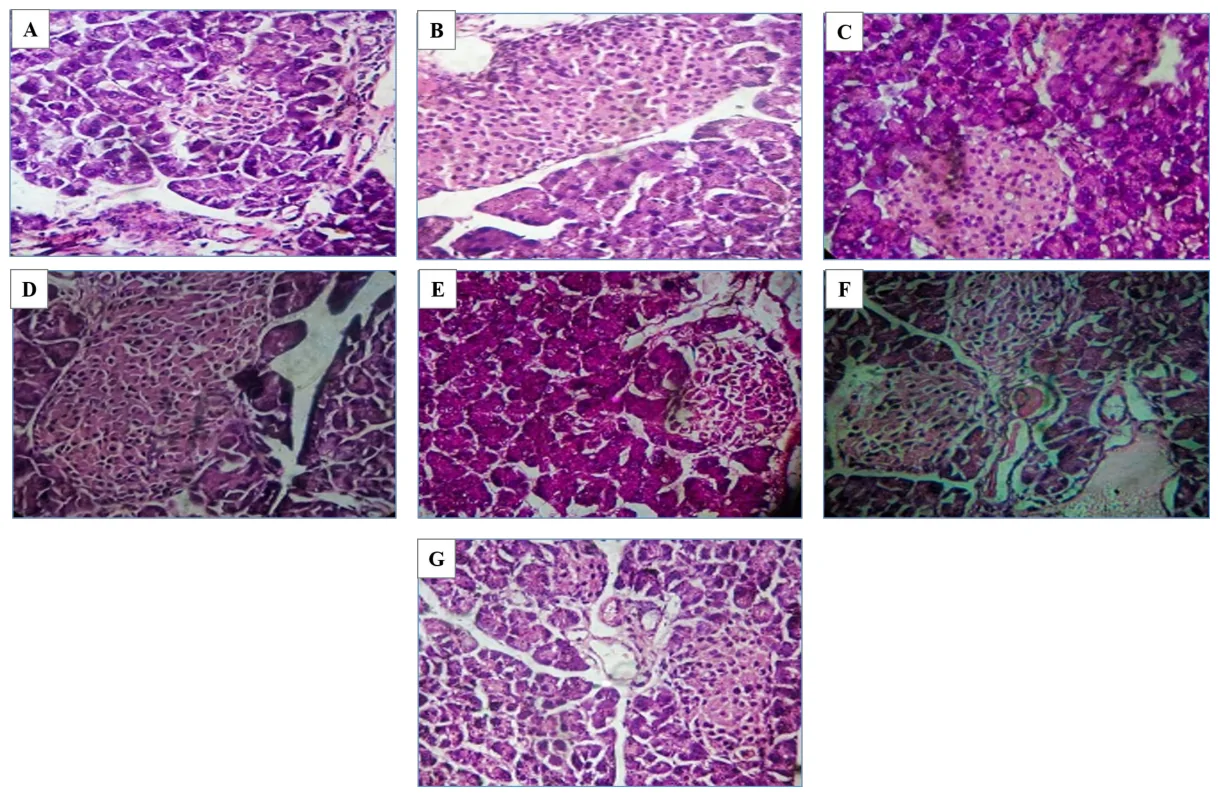
Photomicrographs (400×) of histological sections of the pancreas with haematoxylin and eosin (H&E) staining. (**A**) NC Group: normal islet architecture with densely packed endocrine cells, (**B**) PC Group: atrophic islets with reduced cellularity, vacuolization, and cytoplasmic degranulation, (**C**) SC group: moderately preserved islets architecture, (**D**) LD PE group: modest improvement in islet morphology, (**E**) PE + Probiotic group: improved islet architecture with increased cellularity, (**F**) HD PE group: well preserved islets with organized cellularity, and (**G**) Probiotic group: partial restoration of islet architecture.

**Figure 6:**
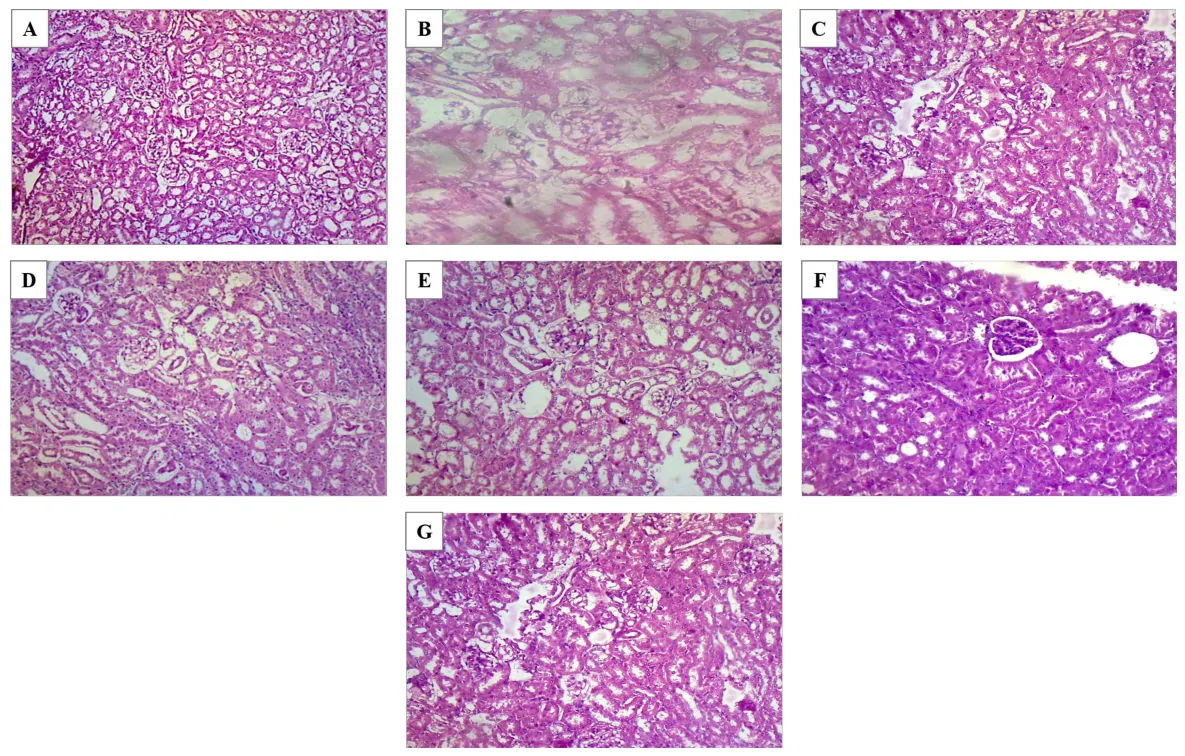
Photomicrographs (100×) of histological sections of the kidney with H&E staining. (**A**) NC Group: normal glomerular and tubular architecture, (**B**) PC Group: evident glomerular and tubular damage, (**C**) SC group: moderately preserved renal architecture, (**D**) LD PE group: mild improvement in glomerular and tubular morphology as compared to PC group, (**E**) PE + Probiotic group: noticeably improved renal morphology and minimal inflammatory changes, (**F**) HD PE group: well-preserved glomerular and tubular structures with organized cellular arrangement, and (**G**) Probiotic group: partial restoration of renal architecture with moderately preserved glomeruli and tubules, though less organized than the HD PE + Probiotic groups.

**Figure 7:**
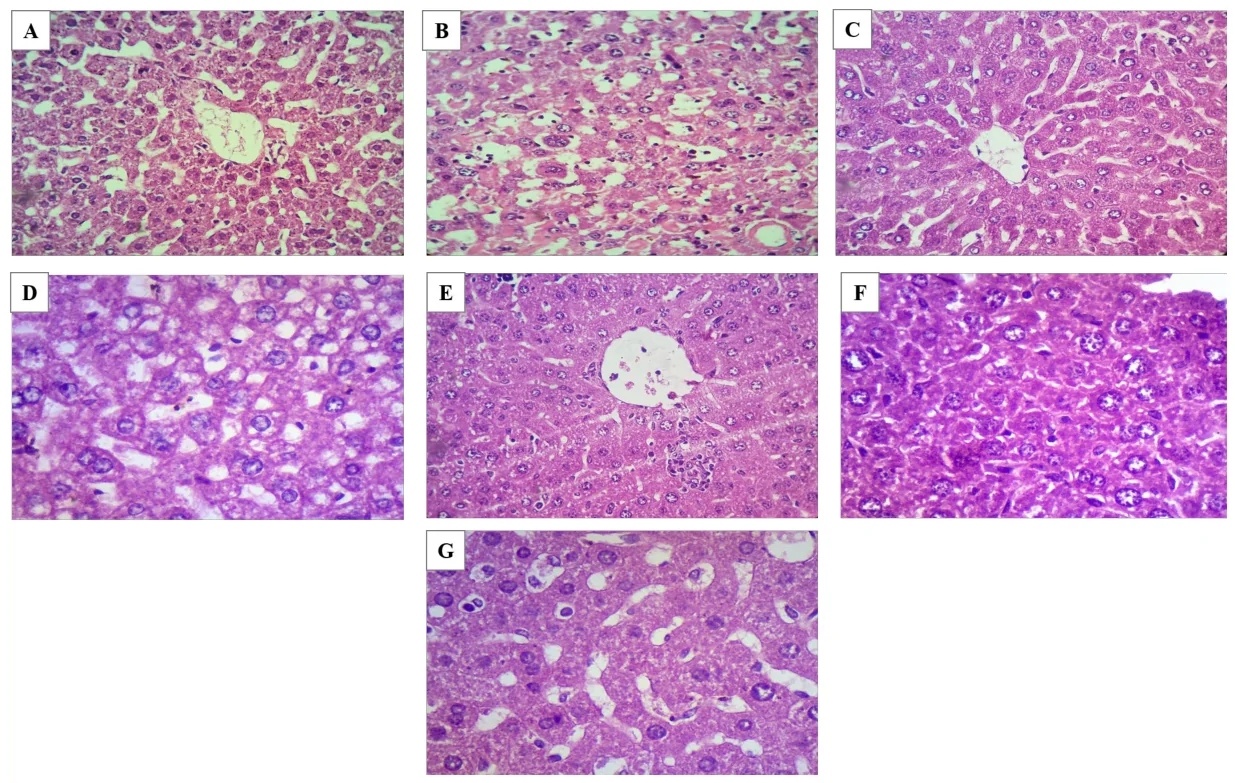
Photomicrographs (400×) of histological sections of the liver with H&E staining. (**A**) NC Group: normal hepatic architecture with well-organized hepatic cords, healthy hepatocytes with distinct nuclei, (**B**) PC Group: marked hepatocellular damage, disorganized hepatic cords and inflammatory cell infiltration, (**C**) SC group: moderately preserved hepatic architecture with mild vacuolization and reduced inflammatory changes, (**D**) LD PE group: mild improvement in hepatocellular morphology with reduced vacuolization, (**E**) PE + Probiotic group: noticeably improved hepatic architecture with well-preserved hepatocytes, (**F**) HD PE group: well preserved hepatic architecture with organized hepatic cords, improved hepatocytes and minimal vacuolization, and (**G**) Probiotic group: partial restoration of hepatic architecture and with moderately preserved hepatocytes.

Histological analysis involves the examination of the arrangement of cells, the size of endocrine cells, cellular density, and vacuolization. The histological section of the NC group showed the pancreas with high cellularity of the islets of Langerhans (Figure 5). However, highly reduced cellularity of the endocrine islets with vacuolization, atrophy, and cytoplasmic degranulation was seen in the PC group. A comparatively regular arrangement of islets was observed in the SC group. The LD PE group produced a modest improvement in pancreatic islet morphology compared with the untreated groups. In the PE + Probiotic group, the islets had more cells with regularly arranged peripheral islet cells. In the HD PE group, preservation of pancreatic islets, increased cellular organization, and reduced vacuolization were observed compared with the untreated diabetic group. Partial restoration of pancreatic tissue was observed in the group treated with *E. ratti*, but cellularity was lower than in the other treated groups. Moreover, H&E-stained sections of kidney (Figure 6) and liver (Figure 7) showed noticeable structural improvements in the treated groups compared with the untreated diabetic groups.

Histomorphometric parameters of the pancreatic islets of the control and treated groups also showed significant results (Table 1).

## 3. Discussion

DM, a severe chronic disorder, causes high blood glucose levels. It is considered an epidemic and is believed to be one of the diseases that has a high mortality rate in adults worldwide [42]. The healthcare system still faces challenges in controlling diabetes with medications that have no side effects. As a result, the need for natural products with minimal side effects and antidiabetic action has increased. Plant extracts and bacterial strains have been reported to preserve pancreatic tissue integrity, reduce oxidative stress, improve insulin secretion, and enhance glucose homeostasis by multiple biological pathways. Plant extracts stimulate insulin release from β cells by activating insulin receptors.

The in vitro assays used in this study were preliminary screening approaches rather than mechanistic investigations. These assays do not fully reproduce the complexity of mammalian glucose metabolism or tissue-specific glucose utilization. Moreover, these assays should be interpreted as supportive evidence for selecting the most effective plant and probiotic strain. The screened plant and LAB strain were further confirmed using an alloxan-induced diabetic mouse model, in which improvement in fasting blood glucose, serum biochemical markers, and pancreatic, liver and kidney histology provides stronger evidence of their therapeutic potential.

In vitro, estimating non-enzymatic Hb glycosylation is an important way to know the mechanism of diabetic effects and their consequences. Although this assay reflects one pathological consequence of chronic hyperglycaemia rather than glucose homeostasis itself, it provides useful preliminary information regarding the antiglycation potential of plant-based bioactive compounds. This study demonstrates selected medicinal plants’ inhibitory role in the Hb glycosylation. The high inhibition of Hb glycosylation by *C. officinalis*, *A. vesica*, and *W. tinctoria* suggests the presence of such compounds that can compete with glucose for binding sites on Hb, thereby reducing the advanced glycation end-product (AGE) formation and reducing diabetic complications including nephropathy. The antiglycation potential of *C. officinalis* as reported previously [43] supports the validity of our screening approach.

The yeast glucose uptake assay is a rapid preliminary screening method for glucose utilization potential of plant extracts. Yeast cells possess different glucose transport systems than mammalian insulin-regulated glucose transporters. Therefore, this assay provides an indirect estimate of glucose utilization and should not be interpreted as evidence of insulin mimic activity. In this study, this assay was used solely to compare the relative glucose utilization capacities of different plant extracts before in vivo evaluation. Recent studies show that glucose transport takes place through stereospecific membrane carriers. The enhanced glucose utilization by yeast cells in the presence of *Z. jujuba*, *W. tinctoria*, and *S. indica* suggests that these extracts may facilitate the transport. These findings are consistent with Nair et al. [44], who reported enhanced glucose uptake by yeast cells (78.42%) with an *Artocarpus heterophyllus* extract.

The bioactive phytochemicals in plant methanolic extracts can bind to glucose. Although the glucose adsorption assay may provide indirect evidence of phytochemicals to reduce glucose availability, it does not accurately reflect intestinal glucose absorption under physiological conditions. Therefore, this assay was only used as a comparative screening tool to screen out the most suitable extract prior to evaluation in hyperglycaemic mouse model. The current study used increasing concentrations of glucose (20 mM, 50 mM, and 100 mM), to investigate the glucose-binding capacity.

α-Amylase catalyses the hydrolysis of α-1,4-glucosidic linkages of starch, glycogen, and various oligosaccharides, and α-glucosidase further breaks down the disaccharides into simpler sugars, readily available for intestinal absorption [45]. The inhibition of their activity in the digestive tract is considered effective in controlling diabetes by diminishing the absorption of glucose produced from the starch by these enzymes. The phytochemicals present in the *S. indica* (leaves)*, A. vesica*, and *C. officinalis* can inhibit α-amylase activity up to 90% as compared to synthetic drugs and other methanolic extracts, suggesting that they might be effective in minimizing glucose availability and significant effect in maintaining postprandial glucose concentration. This is also evidence that the plant extracts may be used as oral agents to manage hyperglycaemia [46].

Recent studies have shown that microbes are crucial for managing diabetes. During in vitro trials, *E. ratti* showed better glucose absorption capacity than other bacterial strains used to estimate the amount of remaining glucose in the supernatant after incubation. The current study demonstrated the in vivo effect of *W. tinctoria* seeds and probiotic on diabetic-induced mice, following screening of the plants and various bacterial strains. Alloxan monohydrate is a diabetogenic agent and can induce oxidative stress. After entering β cells, it undergoes redox cycling that generates ROS. Beta cells, having weak anti-oxidant defence, are vulnerable to oxidative damage [47]. This method is less expensive and extensively used in experimental research to induce diabetes in mice. It acts on the pancreas’s beta cells, partially damaging them and ultimately impairing the amount and quality of insulin they produce. In diabetes, oxidative stress increases in the body, elevating the level of ROS in the islets of Langerhans. This, in turn, reduces antioxidant defence activity and increases free-radical production.

During in vivo evaluation, the progressive reduction in FBG and the recovery of body weight in treated groups suggest that *W. tinctoria* and *E. ratti* alone and in combination may preserve the pancreatic, hepatic and renal morphology in alloxan-induced diabetic mice. The combination group (PE + Probiotic) demonstrated enhanced recovery compared to individual treatment groups, suggesting an enhanced effect of the combined treatment. Raj et al. [48] reported that petroleum ether *W. tinctoria* extract had a beneficial effect on carbohydrate metabolism in diabetic rats. The hypoglycaemic effect of the petroleum ether extract of *W. tinctoria* may effectively potentiate insulin activity by increasing insulin’s pancreatic secretion or its release from bound insulin. The plant extracts lowered serum triglyceride levels significantly, as insulin activates the enzyme lipoprotein lipase, which hydrolyses the triglycerides. The total lipid profile in serum (total cholesterol, triglycerides) of alloxan-induced diabetic rats treated with different doses of extract, i.e., 200 or 400 mg/kg, showed a significant reduction compared with diabetic control rats. The preservation of pancreatic islets and increased cellularity in the treated groups, particularly the combination group, suggest a protective effect on pancreatic endocrine tissue. This may result from the antioxidant activity of the *W. tinctoria*, which could reduce alloxan-induced oxidative damage to the pancreas, or from *E. ratti*-mediated immunomodulation.

## 4. Materials and Methods

### 4.1. Plant Collection and Preliminary Processing

Selected parts of regionally medicinally important plants were collected from different parts of the Lahore region and the botanical garden of the University of the Punjab, viz., leaves of *A. vesica*, flowers of *C. officinalis*, leaves and bark of *S. indica*, seeds of *W. tinctoria* (commercially purchased from a local market and authenticated by the taxonomist at Sultan Ahmad Herbarium, Government College University (GCU) Lahore, with a voucher specimen deposited as GCUSAH-5286), and leaves of *Z. jujuba*. The plant parts were washed, shade-dried, and powdered using a grinder. A weighed amount of powder (20 g) was added to HPLC-grade methanol (200 mL), and the mixture was subjected to a refrigerated shaking incubator at 25 ± 2 °C with a shaking speed of 120 rpm for 24 h. Then, the mixture was filtered using Whatman No. 1 filter paper. Filtrate was subjected to a rotary evaporator at 60 °C under reduced pressure to remove the solvent completely [37]. The extraction was performed repeatedly with yield of 8–10% each time. The thick paste prepared by the rotary evaporator was stored at −20 °C until further use. For in vitro and in vivo assays the dried extracts were reconstituted in DMSO (100 mg/mL) and were further diluted in the appropriate assay buffer to achieve the final working concentration.

### 4.2. Culture of Probiotic Strain

Selected strains of LAB including *Lactobacillus brevis* and *E. ratti* (accession number MF183967 originally isolated from herbivores and characterized by Riaz [38], obtained from the culture stored in the laboratory [38]), were cultured in de Man, Rogosa and Sharpe (MRS) broth medium. Bacterial cells were purified from broth media by washing with distilled water, followed by centrifugation at 10,000 revolutions per minute (rpm) for 10 min. The solution containing the probiotic strain was adjusted to an optical density (OD_600_) of 1, corresponding to 10^11^ colony-forming units (CFUs).

### 4.3. In Vitro Screening Through Biochemical Tests

In vitro assessment of different parts of selected medicinal plants and bacterial strains for hypoglycaemic potential was assayed by using various biochemical assays. The selected in vitro assays were performed sequentially as an initial screening to compare the relative anti-hyperglycaemic potential of different medicinal plant extracts and bacterial strains. Since each assay evaluates a different biological aspect, the combined results were used to identify the most promising candidate for further evaluation.

#### 4.3.1. Inhibitory Activity of Plant Extracts on Hb Glycosylation

Non-enzymatic Hb glycosylation was assayed by a standard protocol. Glucose (2%), Hb (0.06%) and gentamicin (0.2%) solutions were prepared in 0.01 M phosphate buffer separately and mixed in equal ratios (1 mL each). Amounts of 1 mL of different concentrations of methanolic plant extracts were also mixed with a mixture of solutions. All the test tubes were incubated for 72 h in darkness; the glycosylated Hb in the incubated solution was measured at 520 nm by a UV-visible spectrophotometer. The percentage of inhibition was estimated as:

$$\mathrm{Percentage}\;\mathrm{inhibition}\;\mathrm{of}\;\mathrm{glycosylated}\;\mathrm{Hb}\;=\;\frac{A_{c}-A_{s}}{A_{c}}\;\times 100$$

Absorbance of the sample and control were labelled as ‘As’ and ‘Ac’ respectively.

#### 4.3.2. Uptake of Glucose by Eukaryotic Cells (Yeast)

To determine the glucose uptake by eukaryotic cells (yeast), a 10% *v*/*v* suspension was prepared in distilled water. A methanolic extract and a standard drug (1 mL) at increasing concentrations of 100 to 500 µg/mL were added to 20 mM glucose solution (1 mL) and incubated at 25 °C for 10 min. Yeast cells (100 μL) were mixed in the solution and incubated at 37 °C for 60 min, then centrifuged at 2500 rpm for 5 min. After centrifugation, the supernatants were used to measure the glucose in the solution using calorimetric analysis at 500 nm. Inhibition of glucose uptake by yeast cells was estimated as:

$$\mathrm{Percentage}\;\mathrm{of}\;\mathrm{inhibition}\;\mathrm{of}\;\mathrm{glucose}\;\mathrm{uptake}\;\mathrm{by}\;\mathrm{yeast}\;\mathrm{cells}\;=\;\frac{A_{c}-A_{s}}{A_{c}}\;\times 100$$

#### 4.3.3. α-Amylase Inhibitory Assay

In this assay, 1 mL of starch solution (1% *w*/*v*), 1 mL of acarbose solution in the control group, and 1 mL of plant extract in the experimental groups with increasing concentrations (100 and 200 mg/mL), α-amylase solution (1% *w*/*v*), and 2 mL of acetate buffer (0.1 M at pH 7.2) were mixed. A blank control containing all components except α-amylase was used to avoid potential colorimetric interference. An iodine–iodide indicator (0.1 mL) was added to the mixture, followed by incubation for 60 min. A UV-visible spectrophotometer measured the mixture absorbance at 565 nm. The percentage of inhibition was estimated by the following formula:

$$\mathrm{Percentage}\;\mathrm{inhibition}\;\mathrm{of}\;\alpha \text{-}\mathrm{amylase}\;\mathrm{activity}\;=\;\frac{E_{c}-E_{s}}{E_{c}}\;\times 100$$

Absorbance of enzyme activity in the sample and control is labelled as Es and Ec.

#### 4.3.4. Binding Affinity of Glucose to Plant Extract

The bioactive compounds in plant extracts have glucose-binding affinity, so the glucose-binding capacity of methanolic plant extracts was determined. Glucose solutions at different concentrations (20 mM, 50 mM, and 100 mM) and plant extract (1%) were prepared in autoclaved water and DMSO, respectively. Then, 1 mL of each solution was mixed and stirred well. The mixed solution was centrifuged at 3000 rpm for 20 min. The glucose content in the supernatant was determined before incubation and after six hours of incubation and labelled as G1 and G6, respectively. The glucose content in the supernatant was measured calorimetrically at 500 nm. Acarbose was taken as standard.

$$\mathrm{Glucose}\;\mathrm{bound}=\frac{G1-G6}{Weight\;of\;the\;sample}\;\times \;volume\;of\;the\;solution$$

#### 4.3.5. Glucose Consumption by Lactic Acid Bacteria (LAB)

Six strains of LAB, including *L. brevis* and *E. ratti*, were used to evaluate their glucose consumption ability. A glucose solution (1 mL) and 0.1 mL of freshly prepared bacterial cells (CFU 10^11^) were incubated for 10 min at 37 °C. The mixture was centrifuged at 3000× *g* for 5 min. Glucose contents were estimated in supernatants calorimetrically at 400 nm using a UV-visible spectrophotometer:

Glucose utilization = Glucose conc. (before incubation) − Glucose conc. (after incubation).

#### 4.3.6. Colony Rearing for In Vivo Assessment

In the current study, 8-week-old male Swiss albino mice (*Mus musculus*) weighing 22–25 g were reared at the animal house of the Institute of Zoology, University of the Punjab, under standard conditions (25 ± 2 °C; 45–65% humidity) with a 12 h light/dark cycle. They were reared in plastic cages of 7, 5 and 10 inches in height, width and length, respectively. They were fed surplus rodent feed and provided with distilled water ad libitum [38].

#### 4.3.7. Diabetogenesis in Mice

At the experiment’s start, the experimental mice’s blood glucose levels were measured with an electronic glucometer (Accu-chek^®^ Advantage, Roche Diagnostics, Austria, Germany). Alloxan monohydrate (CAT A7413-10G, Sigma–Aldrich, Tauvkirchen, Germany) solution (150 mg/kg body weight) prepared in 0.9% sterile saline was injected intraperitoneally to induce diabetes [49]. After a week of diabetes induction, blood glucose levels were determined by pricking the mice’s tails. Mice with blood glucose ≥250 mg/dL were selected for further experiments [50]. After confirming hyperglycaemia, the animals were randomly allocated to the respective experimental groups. Each mouse was considered an independent biological replicate throughout the study.

### 4.4. In Vivo Experimental Set-Up

Male albino mice weighing 22 to 25 g were divided into seven groups randomly, and each group contained five mice (*n* = 5). The sample size was selected based on commonly used group sizes in exploratory pharmacological studies and in accordance with the principles of 3Rs (replacement, reduction and refinement) which recommend minimizing animal use while maintaining scientific validity. Group I: normal control (NC) comprised non-diabetic mice. Group II: positive/diabetic control (PC), with diabetes induced by alloxan without treatment, was given 0.1 mL normal saline prepared in DMSO (1% *v*/*v*). Group III: standard/acarbose control (SC), comprising alloxanized diabetic mice, was given the standard drug acarbose at a dose of 10 mg/kg body weight prepared in DMSO. Group IV: low dose of plant extract (LD PE) was given methanolic extract of *W. tinctoria* at a dose of 200 mg/kg body weight. Group V: plant extract + bacterial strain (PE + Probiotic) was given a combination of *W. tinctoria* (200 mg/kg body weight) and *E. ratti* (60 µL whose OD_600_ was adjusted at 1). Group VI: high dose of plant extract (HD PE) was given plant extract at a dose of 400 mg/kg body weight. Group VII: bacterial strain (Probiotic) was given *E. ratti* at a dose of 60 µL (OD adjusted at 1). All the doses were given to male albino mice for 21 consecutive days through the oral route using a butterfly needle.

### 4.5. Determination of Body Weight and Fasting Blood Glucose Level

The body weight of the control and experimental groups was measured at regular intervals using an electronic weighing balance. The FBG levels of all groups were determined after 16 h of fasting (when they were allowed free water access) using a digital glucometer (Accu-chek^®^ Advantage, Roche Diagnostic, Germany).

### 4.6. Histological and Biochemical Analysis

On the 21st day, the mice were anaesthetized using ketamine (80 mg/kg) administered intraperitoneally. Direct heart puncture was done to collect the blood (0.8–1.0 mL) using a 1 mL disposable syringe. For serum collection, blood samples were allowed to clot at room temperature for 30 min. The clotted blood was centrifuged at 3000 rpm for 5 min to separate serum. The supernatant (serum) was carefully aliquoted into fresh Eppendorf tubes and stored at −20 °C until further analyses. After blood collection, pancreatic, liver and kidney tissue excised from dissected animals, washed with ice-cold phosphate-buffered saline (pH 7.4), fixed in formalin (10%) for 24 h at room temperature. Following fixation, tissues were dehydrated through a graded ethanol series, cleared in xylene and embedded in paraffin wax. Serial sections of 5 µm were cut using a rotary microtome. The sections were stained with haematoxylin and eosin (H&E) following standard protocols. The prepared slides were ultimately examined under a light microscope using low and high (40–400×) magnification [51,52]. Histomorphometric analysis of pancreatic sections was performed using the commercial program ProgRes^®^ 2.1.1 CapturePro Camera Control Software (Jenoptik, Jena, Germany) and imageJ 1.52a for quantitative measurements. For each animal, five non-adjacent pancreatic sections (3–5 islets per section) were examined. All histomorphometric analyses were performed by an investigator blinded to the experimental group allocation.

The serum was subjected to a renal function test (RFT), including serum creatinine and urea levels, and a liver function test (LFT), such as bilirubin, AST, ALT, and ALP. Lipid profiles were analysed using a kit in according with the manufacturer’s instructions using a Mindray BS-240VET (Mindray, Animal Care, Shenzhen, China) automated veterinary chemistry analyser.

### 4.7. Statistical Analysis

All in vitro experiments were performed as three independent experiments (biological replicates), each with technical triplicate measurements. Data are presented as mean ± standard error of the mean (SEM) (n = 3 independent experiments). Technical replicates were averaged within each independent experiment before statistical analysis. For the in vivo experiments, each mouse represented an independent biological replicate (n = 5 per group). For single time-point measurements (biochemical, histomorphometric parameters), statistical analyses were performed using one-way ANOVA followed by Duncan’s multiple range test (DMRT) for post hoc multiple comparisons using SPSS 16.0 software. All graphs were plotted in Microsoft Office 2018. Differences were considered statistically significant at *p* ≤ 0.05. Different letters above bars indicate statistically significant differences.

## 5. Conclusions, Limitations and Future Recommendations

The results of the present study showed that *W. tinctoria* (seeds extract), and LAB (*E. ratti*) alone and in combination could be effectively used to lower the elevated blood glucose in alloxan-induced hyperglycaemia. The treatment also improved the biochemical markers associated with hepatic and renal functions and preserved pancreatic, renal and hepatic morphology. When different experimental groups were compared with the control group, the HD PE and PE + Probiotic groups showed a significant reduction in serum glucose levels. Histomorphometric analysis of the H&E-stained pancreatic sections and histological evidence of liver and kidney sections showed preserved pancreatic islets and improved histological features of liver and kidney in treated animals compared with the alloxan-induced diabetic group. However, the measurements for endocrine cells represent all cells within the islets of Langerhans, as H&E staining does not permit definitive identification of individual β cells. The restoration of islet cellularity in the HD PE and PE + Probiotic groups correlates well with the observed improvements in the FBG levels, suggesting functional recovery of insulin-producing capacity.

Based on in vitro analyses, the methanolic extract of *W. tinctoria* seeds showed an overall improvement compared all tests, warranting evaluation in an in vivo study. *E. ratti*, which showed the best in vitro results, was chosen for in vivo purposes. These products also showed enhanced effects when combined. Although these natural products showed maximum antidiabetic potential, the underlying mechanisms and factors of diabetes treatment are yet to be discovered.

The present study employed several conventional in vitro biochemical assays as preliminary screening tools to identify promising plant extracts and probiotic strains. Despite their widespread use, these assays do not capture the complexity of mammalian glucose metabolism or elucidate the underlying mechanisms. Future studies should include mechanistic investigations involving insulin signalling pathways, serum insulin measurements and gene expression analyses to further confirm the therapeutic mechanisms of *W. tinctoria* seeds and *E. ratti*. However, additional safety evaluations including genomic characterization and antimicrobial profiling are required before considering a probiotic for clinical or commercial application [37]. Moreover, in vivo studies were conducted with a relatively small sample size (*n* = 5 per group), which was chosen in accordance with ethical guidelines and the principles of 3Rs to minimize animal use. Further studies using larger cohorts are recommended to confirm the reproducibility and generalizability of the present findings. Comprehensive toxicological, pharmacological, and mechanistic studies are required before considering potential clinical applications.

## Figures and Tables

**Table 1:** Histomorphometric parameters of the pancreatic sections of control and treated groups.

Parameters	NC Group	PC Group	SC Group	LD PE Group	PE + Probiotic Group	HD PE Group	Probiotic Group
No. of islets (N/10 mm^2^)	10.33 ± 0.66	7.000 ± 0.00	8.33 ± 0.33	7.00 ± 0.54	9.00 ± 0.55	7.00 ± 0.57	7.00 ± 0.58
Diameter of islets (µm)	148.97 ± 10.09	127.59 ± 9.97	177.93 ± 14.24	133.52 ± 15.62	155.70 ± 19.60	156.06 ± 10.61	104.18 ± 10.68
No. of endocrine cells per islet (cells/islet)	6.67 ± 0.34	4.00 ± 0.00	4.66 ± 0.33	4.00 ± 0.57	6.33 ± 0.88	5.00 ± 1.00	5.00 ± 0.57
Diameter of endocrine cells per islet (µm)	17.35 ± 2.43	15.78 ± 2.68	16.82 ± 2.63	17.79 ± 2.41	16.65 ± 2.14	18.06 ± 2.67	17.44 ± 2.11
Volume of islets (µm^3^)	1.58 × 10^6^ ± 3.52 × 10^7^	1.18 × 10^6^ ± 2.81 × 10^5^	3.19 × 10^6^ ± 8.10 × 10^5^	1.25 × 10^6^ ± 1.18 × 10^6^	1.98 × 10^6^ ± 5.57 × 10^5^	2.64 × 10^6^ ± 5.23 × 10^5^	5.99 × 10^5^ ± 2.48 × 10^6^
Volume of endocrine cells (µm^3^)	1.89 × 10^4^ ± 1.23 × 10^3^	8.24 × 10^3^ ± 1.42 × 10^3^	1.18 × 10^4^ ± 1.33 × 10^3^	1.16 × 10^4^ ± 1.28 × 10^3^	1.53 × 10^4^ ± 9.32 × 10^3^	1.54 × 10^4^ ± 1.44 × 10^3^	1.39 × 10^4^ ± 1.04 × 10^3^

## Data Availability

The original contributions presented in this study are included in the article/Supplementary Materials. Further inquiries can be directed to the corresponding author.

## Supplementary Materials

The following supporting information can be downloaded at: https://www.mdpi.com/article/10.3390/ijms27198792/s1, Figure S1: HPLC chromatoram of methanolic extract of *W. tinctoria* seeds; Figure S2: FT-IR spectrum obtained from the methanolic extracts of *W. tinctoria* seeds showing different peaks between wavelengths 4000 to 500 cm^−1^; Table S1: Significant peaks and their molecular assignments based on the FT-IR analysis of methanolic extracts of *W. tinctoria* seeds. References cited in Supplementary Materials [53,54,55].
