# Supplementary material for: Anti-Hyperglycaemic, Hepatoprotective, and Nephroprotective Effects of Wrightia tinctoria Seeds and Enterococcus ratti in Alloxan-Induced Diabetic Mice
Source: Int J Mol Sci. 2026 Sep 30;27(19):8792. doi: 10.3390/ijms27198792 (PMC13665475; doi:10.3390/ijms27198792)
Supplement: Supplementary file 1 [file ijms-27-08792-s001.zip › ijms-4540263-supplementary.pdf]

Supplementary Files

Anti-hyperglycaemic, hepatoprotective, and nephroprotective effects of *Wrightia tinctoria* seeds and *Enterococcus ratti* in alloxan-induced diabetic mice

Supplementary Files: HPLC and FT-IR analyses of *Wrightia tinctoria* seeds

Paper title: Anti-hyperglycaemic, hepatoprotective and nephroprotective effects of *Wrightia tinctoria* and *Enterococcus ratti* in alloxan-induced diabetic mice

Figure S1: High-Performance Liquid Chromatography (HPLC) profiling of methanolic extract of *W. tinctoria* seeds.

Methodology:

1. Sample preparation

Approximately 10 ± 0.01 mg of lyophilized seed powder was weighed on an analytical balance. The seed powder was dissolved in HPLC-grade methanol to prepare a 1 mg/mL stock solution. The mixture was vortexed for 2 minutes, then briefly sonicated for 5 minutes at room temperature to ensure complete dissolution. The initial mobile phase composition was further diluted to bring the concentration within the calibration range. After dilution, the solution was filtered through a 0.22 μm PVDF syringe filter into an amber HPLC vial immediately before injection.

2. Calibration standard preparation

A separate, independently sourced reference standard (Acrylamide and Quercetin) (⩾98% purity) was prepared at 1 mg/mL in HPLC grade methanol as stock solution. From this stock, eight working standards were prepared at concentrations of 0.1, 0.5, 1, 5, 10, 25, 50, and 100 μg/mL, diluted in the mobile phase. Optionally, an internal standard was included at a fixed concentration across all standards and samples to correct for injection-to-injection variability.

3. Column and detector setup

A reverse-phase C18 column (4.6 × 250 mm, 5 µm particle size) was used. The column oven temperature was set to 30°C. UV detection was performed at 254 nm; when a diode-array detector (DAD) was available, full spectra from 200 to 400 nm were collected to confirm peak purity. The column was equilibrated with the initial mobile phase composition for at least 20 minutes before the first injection.

4. Mobile phase and gradient program

Mobile phase A consisted of ultrapure water with 0.1% formic acid (v/v), and mobile phase B consisted of HPLC-grade acetonitrile with 0.1% formic acid (v/v). Both phases were degassed by sonication or using an inline degasser. The following gradient program was run at a flow rate of 1.0 mL/min:

Time (min)	% B
0–2	5
2–18	5 → 35 (linear ramp)
18–22	35 → 90 (column wash)

5. Injection sequence

A 10 µL aliquot was injected per run in the following order: solvent blank (methanol), calibration standards from lowest to highest concentration, quality control (QC) mid-point standard, sample replicates (minimum of triplicate), and finally a QC mid-point standard again to monitor system drift. All samples were kept at ambient autosampler temperature; when possible, samples were maintained at 10°C in the autosampler tray to minimize degradation of thermolabile compounds.

6. Peak identification

Peaks were identified by comparing their retention times against those of authentic reference standards. Using the DAD, the UV spectrum of the sample peak was overlaid with that of the standard; spectra were required to match within accepted thresholds (purity angle < purity threshold). Any deviation indicated possible co-elution or degradation products.

7. System suitability and reporting

Before reporting results, system suitability was confirmed from the reference standard chromatogram. The following criteria were required: tailing factor ≤ 2.0, theoretical plate count ≥ 2000, and relative standard deviation (RSD) of replicate injections ≤ 2%. Results were reported with the raw chromatogram, calibration curve (including equation and R^2 value), peak table (retention time, area, tailing factor), and the calculated compound content with standard deviation across replicates (Baskaran *et al*., 2025).

Results:

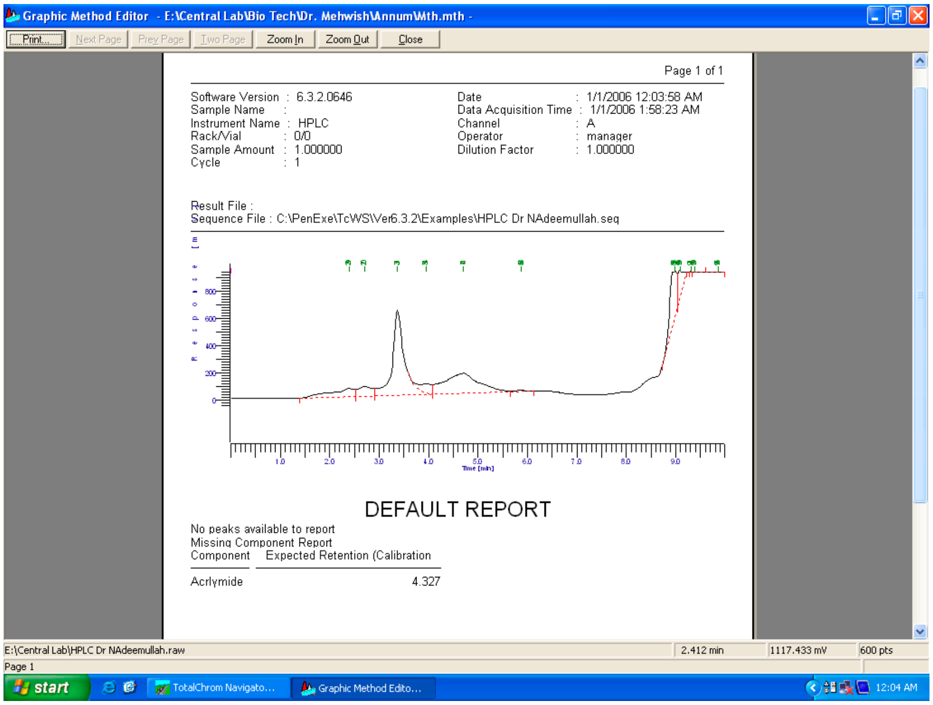

Figure S1: HPLC chromatoram of methanolic extract of *W. tinctoria* seeds.

A peak was observed at 2.412 minutes with a signal intensity of 1117.3 mV, followed by a peak after the eighth minute. This peak confirmed the presence of highly polar and hydrophilic compounds. The known antidiabetic compounds from *W. tinctoria* are flavonoids, such as quercetin (retention time 8.78 minutes), and phenolic acids, like chlorogenic acid, which usually elute between 8th and 25th minutes under standard C18 gradient conditions. Based on the Data, the possible compound classes are polar sugars, small organic acids (citric, malic, or oxalic acid), and amino acids / small peptides. HPLC data therefore provide preliminary information on the polarity of the major compounds present but do not constitute definitive compound identification or quantification.

Figure S2: Fourier Transform Infrared (FT-IR) Spectroscopy:

Methodology:

The functional groups present in methanolic extracts of W. tinctoria seeds were identified by Fourier Transform Infrared (FTIR) spectroscopy (Nicolet 6700, Summit lite, Thermo Scientific™). All spectra were recorded in transmittance mode over the wavenumber range of 4000 to 500 cm^{-1} with a resolution of 4 cm^{-1} (Baskaran *et al.*, 2025; Hemavathy et al., 2019).

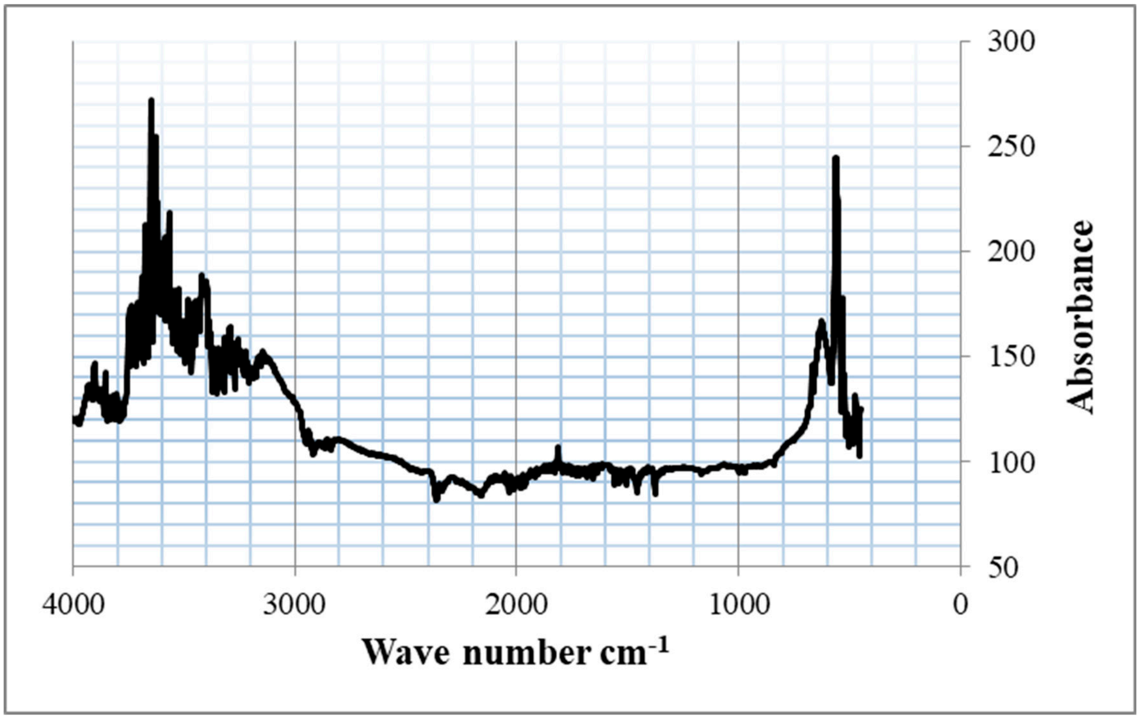

Figure S2: FT-IR spectrum obtained from the methanolic extracts of *W. tinctoria* seeds showing different peaks between wavelengths 4000 to 500 cm^{-1}.

Analysis interpretation:

FT-IR analysis of methanolic extracts of *W. tinctoria* seeds shows several dominant broad and sharp peaks as given in Table S1.

Table S1: Significant peaks and their molecular assignments based on the FT-IR analysis of methanolic extracts of *W. tinctoria* seeds.

Wavenumber	Peak shape &	Functional group	Possible chemical class

(cm⁻¹)	intensity		
**~3390**	Very Broad, Strong	O-H stretching (H-bonded)	Phenolic compounds, Flavonoids, Carbohydrates, Glycosides
**~2922**	Strong, Sharp	C-H stretching (asymmetric & symmetric of $–CH_2$ & $-CH_3$)	Aliphatic compounds, Terpenoids, Steroids, Fatty acids
**~1710**	Moderate, Sharp	C=O stretching (ester/acid)	Carboxylic acids, Coumarins, Xanthones, Terpenoids
**~1610**	Moderate, Sharp	C=C aromatic ring stretching, C=O conjugated	Aromatic compounds, Flavonoids, Phenolics
**~1510**	Moderate	C=C aromatic ring stretching (in-plane)	Aromatic ring of flavonoids and phenolics
**~1450 & 1375**	Moderate	C-H bending ($-CH_3$, $-CH_2-$)	Aliphatic groups (terpenoids, steroids, fatty acids)
**~1240-1260**	Strong, Broad	C-O stretching (aryl-alkyl ether, phenolic OH)	Flavonoid glycosides, Phenolic acids,
**~1040-1060**	Very Strong, Broad	C-O-C stretching (glycosidic linkage) + C-OH bending	Carbohydrates, Glycosides

The FT-IR spectrum indicates a rich phytochemical profile, dominated by polyphenols, flavonoids, and glycosides (at 1040 cm^{-1}, an abundant C-O-C glycosidic linkage that inhibits α-amylase), polyphenols (which improve insulin sensitivity and glucose uptake), and carbohydrates (Subramanian *et al*., 2005).

References:

Baskaran, V. M.,Muthupriya, S., Manobala, S., Hemanathi, M., & Ragul Raj, T. (2025). Phytochemical Estimation and In Vitro Antihyperglycemic and Antioxidant Study of Leaf Extract of *Wrightia tinctoria* (Roxb) R.BR. *Saudi Journal of Biomedical Research*, 10 (7), 245–255.

Subramanian, K., Kumar, P. S., Jeyapal, P., & Venkatesh, N. (2005). Characterization of ligno-cellulosic seed fibre from *Wrightia Tinctoria* plant for textile applications—an exploratory investigation. *European Polymer Journal*, *41*(4), 853–861.

Hemavathy, A., Shanthi, P., Sowndharya, C., Thiripura, S. S., & Priyadharshni, K. (2019). Extraction and isolation of bioactive compounds from a therapeutic medicinal plant-*Wrightia tinctoria* (Roxb.) R. *Br Int J Pharmacogn Phytochem Res*, *11*(3), 199-204.
